# The three-dimensional kinematics and spatiotemporal parameters of gait in 6–10 year old typically developed children in the Cape Metropole of South Africa – a pilot study

**DOI:** 10.1186/s12887-016-0736-1

**Published:** 2016-12-03

**Authors:** Yvonne Smith, Quinette Louw, Yolandi Brink

**Affiliations:** Division of Physiotherapy, Department of Interdisciplinary Health Sciences, Faculty of Medicine and Health Sciences, Stellenbosch University, South Africa, PO Box 241, Cape Town, 8000 South Africa

**Keywords:** 3D gait analysis, Walking, Children, Spatiotemporal parameters, Kinematics

## Abstract

**Background:**

Functional gait is an integral part of life, allowing individuals to function within their environment and participate in activities of daily living. Gait assessment forms an essential part of a physical examination and can help screen for physical impairments. No three-dimensional (3D) gait analysis studies of children have been conducted in South Africa. South African gait analysis laboratory protocols and procedures may differ from laboratories in other countries, therefore a South African data base of normative values is required to make a valid assessment of South African children’s gait. The primary aim of this study is to describe joint kinematics and spatiotemporal parameters of gait in South African children to constitute a normative database and secondly to assess if there are age related differences in aforementioned gait parameters.

**Methods:**

A descriptive study was conducted. Twenty-eight typically developing children were conveniently sampled from the Cape Metropole in the Western Cape, South Africa. The 3D lower limb kinematics and spatiotemporal parameters of gait were analyzed. The lower limb Plug-in-Gait (PIG) marker placement was used. Participants walked bare foot at self-selected speed. Means and standard deviations (SD) were calculated for all spatiotemporal and kinematic outcomes. Children were sub-divided into two groups (Group A: 6–8 years and Group B: 9–10 years) for comparison.

**Results:**

A significant difference between the two sub-groups for the normalized mean hip rotation minimum values (*p* = 0.036) was found. There was no significant difference between the sub-groups for any other kinematic parameter or when comparing the normalized spatiotemporal parameters.

**Conclusion:**

The study’s findings concluded that normalized spatiotemporal parameters are similar between the two age groups and are consistent with the values of children from other countries. The joint kinematic values showed significant differences for hip rotation, indicating that older children had more external rotation than younger children.

## Background

Humans walk an average of 10 000 steps per day [1]. Functional gait forms an integral part of life, allowing individuals to function within their environment and participate in activities of daily living. The importance of locomotion from a psychosocial point is often overlooked. It facilitates normal social interaction and participation in recreational activities [2, 3]. The ability to walk is one of the critical elements in measuring and improving quality of life and reflects the individual’s health status [2, 4].

Gait assessment is part of the physical examination and can help screen for a range of physical impairments and abnormalities [5]. Similarly, analysis of gait at an early age can help predict motor outcome in cerebral palsy [6]. Evidence shows that a better understanding of normal development may be useful in interpreting abnormal findings [7, 8]. Gait analysis can also be used as an outcome measure to evaluate the effect of an intervention such as the single event multi-level surgery for children with cerebral palsy [9].

Although gait analysis has been conducted in children since the 1980’s, surprisingly little is known about age related gait patterns in children with typical development [10]. The gait data of 85 healthy children (4–16 years) at self-selected walking speed were examined using the VICON Plug-In-Gait (PIG) model [11]. Gait cycles of thorax, spine and pelvis kinematics in the sagittal, frontal and transverse planes were recorded, and stratified by age and normalized speed. The sagittal thorax and spine movements were found to be gradually and significantly associated with age, but less so with speed, so that with increasing age, children tended to lean their trunk forward relative to the pelvis. In contrast, the frontal and transverse parameters of spine and pelvic movements were found to be mainly dependent on speed, not age [11]. A 3D motion analysis study of fifty children between 7 and 11 years old using the ZEBRIS CMS 70 P system measured flexion (F), extension (E), abduction (abd) and adduction (add) angles of the hip joint, the F and E of the knee and ankle joints and foot rotations for each age group [12]. Their findings were consistent with other published literature reporting on joint kinematics and suggested that children 7–11 years old presented with adult-like gait patterns [12–14].

Speed strongly influences other spatiotemporal parameters, joint kinematics and kinetics of walking gait in children aged 4–17 years [14–16]. Van der Linden et al. [15] and Schwartz et al. [16] found that kinematics, kinetics and EMG readings corresponded strongly with speed. The kinetic values of peak propulsive forces were found in most of the joints of the lower limb during increased walking speeds, as well as significant differences in the kinematics of ankle dorsiflexion (DF), knee E and hip F and E ranges in the sagittal plane [15, 16]. EMG readings showed greater muscle activity at increased speeds for the hamstrings, rectus femoris and tibialis anterior muscles [15, 16]. In a South African based study of 200 children between the ages of 1 and 13 years, the study reported an increased speed as age increased. The data for children from 4–13 years of age were centred on the data for adults confirming that neuromaturation of gait patterns occurs from four years onwards and the authors concluded that speed is a reliable measure of gait maturation [17].

To the researchers’ knowledge, no studies describing the three-dimensional (3D) gait analysis of South Africa children have been conducted. Currently there exists no normative dataset for the gait parameters of typically developed children in South Africa. A normative database of typically developed South African children will provide a valid reference dataset to determine how gait is affected in children with gait abnormalities due to e.g. cerebral palsy which is highly prevalent in Africa [18]. Furthermore, a South African database of normative values is required to demonstrate that a South African gait analysis laboratory, protocols and procedures compares to international standards. It is known that gait laboratories from different countries have reported variability in gait patterns particularly hip rotation and foot progression angles. These differences could be due to the different marker placement or data processing protocols between laboratories [19]. Ferrari et al. [20] is one of the first studies that compared five different gait analysis protocols to assess inter-protocol variability. Prior to their study there had not been an emphasis on the standardization of gait analysis protocols between different laboratories, thus no gold standard for evaluation of gait. However, recently there has been an increase in studies measuring the reproducibility of data within and between gait laboratories. The primary aim of this study is to describe joint kinematics and spatiotemporal parameters of gait in South African children to constitute a normative database. Secondly we assessed if there are age related differences in the aforementioned gait parameters. We hypothesized that there will be no differences in gait parameters in children 6–8 years old compared to 9–10-year-old children.

## Methods

### Ethical considerations

Approval from Stellenbosch University Human Research Ethics Committee was obtained (S13/10/220). Parents / guardians of participants signed an informed consent form prior to data collection. Participants seven years and older signed an informed assent form once the procedure was explained and all their questions answered.

### Study design and setting

A descriptive study was conducted at the Physiotherapy and FNB 3D Movement Analysis Laboratory, Stellenbosch University, Cape Town, South Africa.

### Population and eligibility

The study population included typically developed boys and girls between the ages of 6–10 years residing within the Cape Metropole of the Western Cape in South Africa. This geographical area was chosen due to easy accessibility. Boys and girls from varied ethnic and socio-economic backgrounds, who attended mainstream schools or education centers and had good general health, were included in the study. Only children ten years and younger were eligible to participate as girls and boys start puberty around the ages of 10–11 and 12 years respectively and this stage is characterized by rapid skeletal growth and physical changes [21].

Children diagnosed with Attention Deficit Hyperactivity Disorder, Cerebral Palsy, Scoliosis, Fetal Alcohol Syndrome, Developmental Coordination Disorder, Duchene’s, hip dysplasia or any similar syndrome by a health care practitioner were excluded. Children with a BMI level >30 were unsuitable for this study and were also excluded [22]. If children sustained a recent (past six months) traumatic injury to the neuro-musculoskeletal system, complained of recurrent idiopathic musculoskeletal pain, or were unwell on the day of testing, they were also excluded from the study as it could potentially influence their normal gait patterns.

### Sampling

Convenient sampling of centers was performed. Researchers approached local crèches (n = 2), after care facilities (n = 4) and primary schools (n = 3) and invited all eligible children to participate in the study. The sample size justification for this study was based on the primary aim i.e. to describe gait parameters of South African children and therefore aimed to include 30 participants as suggested by Billingham et al. [23]. We calculated the margin of error for the sample mean based on an estimated population standard deviation of 2 degrees [19]. At a 95% confidence level, we calculated that the margin of error of the sample mean would be 0.78 degrees if 28 participants are included.

### Measurement instruments

The VICON motion analysis (MX T-series, Vicon Motion Systems Ltd, Oxford, UK) system with eight T-10 Vicon cameras and Nexus 1.4 116 software was used to capture walking trials. Kinematics were calculated per the PIG model [24]. A manual medium international standard goniometer (8") was used to evaluate the joint ranges of the lower limb. The VICON has demonstrated high accuracy and reliability and demonstrated to have less than a 1.5-degree error [25, 26]. An electronic scale was used to measure participants’ weight in kilograms (kg). Height was measured in millimeters (mm) using a T-bar tape measure. Leg length was measured in millimeters (mm), using a measurement tape from predetermined landmarks (anterior superior iliac spine and medial malleoli). A general health and activity questionnaire included questions on previous injuries, general health, as well as the type and frequency of sport the child participated in.

### Study procedure

Once potential participants had been identified for the study, parents / guardians received written information about the study. They also received written informed consent forms and a general health and activity questionnaire to complete. The questionnaire enabled the researchers to screen potential participants for eligibility. Children, who were eligible to participate in the study, were scheduled for gait analysis during April – July 2014.

Participants were dressed in shorts and a sport top so that the anatomical landmarks were exposed. The children were asked to remain bare footed during the physical evaluation, calibration and gait analysis. The researcher conducted a standard physical evaluation on each participant. Each child’s lower limb joint ranges, which included: hip F, E, abd; add, internal rotation (int rot) and external rotation (ext rot); knee F and E; and ankle plantar flexion (PF), DF with knee straight and DF with knee bent, were measured using a medium international standard goniometer (8") to screen for major joint range discrepancies. Height, weight and leg length were measured.

For data capture, the lower limb PIG marker placement was used. The markers were placed by two trained laboratory technicians (on randomly selected days) for whom intra- and inter-person reliability had been established and deemed satisfactory [personal communications QA Louw]. Standard system and subject calibration procedures were performed. The walking procedure was explained to the participants and each had two practice walking trials. Participants were asked to walk the full length of the walkway (±20 m) six times at self-selected speeds. A walking trial was deemed successful if the child did not look around or veered from the walkway.

### Data analysis

A validated numerical optimization method to correct for any displacement of the thigh markers from the true femoral frontal plane were used [27–29]. This method is a functional approach in which the knee axis orientation is estimated based on the assumption of minimum variance in the frontal plane motion of the knee. This ensures that the knee axis estimate, and by implication the hip rotation parameter, is reliable and valid given the reality of soft-tissue-artefacts. Gap filling was performed using the standard Woltring filter supplied by VICON [30]. A validated foot velocity algorithm which detects foot contact and loss of foot contact using foot marker kinematics were used [31]. The events for foot contact and lowest vertical position of the pelvis were calculated automatically using Matlab Version R2012b (Mathworks, Natick, MA, USA). Data was filtered with a 4th-order Butterworth filter at a 10Hz cut-off frequency and segment and joint kinematics were calculated using the PIG-model. Data was exported to Matlab to extract the spatiotemporal parameters and the joint kinematics of the lower limbs. The spatiotemporal parameters were normalized using leg length, according to the following formulae: $\mathrm{step}\ \mathrm{length}\ \left(\mathrm{meter}\right) = \frac{step\  length}{leg\  length}$; $\mathrm{stride}\ \mathrm{length}\ \left(\mathrm{meter}\right) = \frac{stride\  length}{leg\  length}$; $\mathrm{cadence}\left(\mathrm{steps}\ \mathrm{per}\ \mathrm{second}\right)\kern0.5em  = \mathrm{cadence}\ \mathrm{x}\sqrt{\frac{leg\  length}{g}}$; $\mathrm{walking}\ \mathrm{speed}\ \left(\mathrm{meter}\ \mathrm{per}\ \mathrm{second}\right) = \frac{speed}{\sqrt{leg\  length\ x\ g}}$ where *g* refers to the acceleration due to gravity (9.81 ms^-2^) [32, 33].

### Statistical analysis

Descriptive statistics (mean, SD) were used to describe the participants’ demographics and median and ranges were used to describe the outcome measures i.e. joint kinematics and spatiotemporal parameters. The data followed a skewed distribution and thus Mann-Whitney statistical tests were performed to determine significant differences between age groups for spatiotemporal parameters and joint kinematics. The joint kinematics were statistically analysed using the minimum and maximum values. We have conducted a Wilcoxon-Mann Whitney post hoc power analysis using G- Power version 3.1. Considering a significant level of *p*-value was ≤ 0.05, a difference of at least 5 degrees and a standard deviation of 5 degrees (considering the variability in the dataset), the statistical power was calculated to be 70%.

## Results

### Sample description

Twenty-eight children with mean age 8.6 years (±1.3), weight 32.8 kg (±12.4) and height 1.4 m (±0.1) participated in the study. The demographics of the children per age group are shown in Table 1. Eighteen Mix-raced children, seven Black children and three Caucasian children participated in the study.

**Table 1:** The mean values for weight, height and BMI for boys and girls per age group (*n* = 28)

BOYS					
	6 years (*n* = 0)	7 years (*n* = 2)	8 years (*n* = 3)	9 years (*n* = 4)	10 years (*n* = 3)
Weight (kg)	-	36.3	30.3	38.7	46.6
Height (m)	-	1.3	1.3	1.4	1.4
BMI	-	21.4	17.7	19.7	21.2
GIRLS					
	6 years (*n* = 3)	7 years (*n* = 1)	8 years (*n* = 2)	9 years (*n* = 4)	10 years (*n* = 6)
Weight (kg)	26.4	20.1	29.0	38.2	44.8
Height (m)	1.2	1.1	1.4	1.4	1.5
BMI	17.6	16.3	15.9	20.3	21.7

### General health and activity questionnaire

None of the participants had any health problems, presented with developmental delays or motor problems or suffered recent injuries, illnesses or body pain in the past six months. Although all the children participated in sport or a recreational activity, a range of different activity levels were reported. The outcome ranged from playing two types of sport, 4 times a week to one type of sport, once a week.

### Spatiotemporal parameters

The non-normalized spatiotemporal parameters for the whole group were 2.2 (1.85–2.41), 1.26 (1.15–1.55), 0.59 (0.55–0.69) and 1.16 (1.09–1.36) for cadence (steps per second), walking speed (meter per second), step length (meter) and stride length (meter) respectively. For the group, the normalized median values for cadence, speed, step length and stride length were: 0.81 (0.68–0.93), 0.48 (0.44–0.58), 0.83 (0.73–0.96) and 1.64 (1.45–1.90) respectively. There were no differences between boys and girls for the spatiotemporal parameters therefore the genders were combined in each age group. Due to the small sample size, number of participants per age group and no statistical significant differences in the spatiotemporal parameters between the 6–8 year olds and the 9–10 year olds, the five age groups were divided into two groups: Group A (6.0–8.11 year olds) and Group B (9.0–10.11 year olds) [11].

Table 2 presents the non-normalized and normalized median and range values for the spatiotemporal parameters of each age group. Table 3 shows the non-normalized and normalized median and range values for the spatiotemporal parameters for the two age subgroups (Group A and Group B) as well as the *p*-values indicating the statistical significance between the two groups. There was a significant difference between the younger and older children for all the non-normalized parameters. However, this significance did not persist when controlling for height as can be seen by the *p*-values for the normalized mean values.

**Table 2:** Spatiotemporal Parameters (non-normalized and normalized) for each age group

	6 years (*n* = 3)		7 years (*n* = 3)		8 years (*n* = 5)		9 years (*n* = 8)		10 years (*n* = 9)	
	median	range	median	range	median	range	median	range	median	range
	Non-normalized									
Cadence (steps / sec)	2.42	2.25–2.68	2.34	2.18–2.38	2.21	1.98–2.72	2.18	1.85–2.39	2.16	1.93–2.41
Speed (ms^-1^)	1.26	1.09-1.45	1.14	0.88-1.27	1.16	0.90-1.38	1.34	1.15-1.49	1.32	1.18-1.55
Step Length (m)	0.53	0.48–0.56	0.49	0.42–0.56	0.53	0.46–0.60	0.62	0.59–0.66	0.62	0.54–0.69
Stride Length (m)	1.04	0.95–1.11	0.97	0.82–1.11	1.05	0.92–1.17	1.23	1.15–1.32	1.23	1.09–1.36
	Normalized									
Cadence	0.91	0.90–1.09	0.93	0.93–0.95	0.79	0.77–1.11	0.80	0.69–0.93	0.77	0.68–0.92
Speed	0.50	0.44–0.59	0.46	0.38–0.50	0.45	0.36–0.56	0.50	0.44–0.58	0.48	0.44–0.57
Step Length	0.83	0.75–0.90	0.79	0.73–0.86	0.79	0.71–0.85	0.85	0.80–0.96	0.82	0.73–0.87
Stride Length	1.63	1.48–1.79	1.56	1.49–1.72	1.55	1.39–1.69	1.68	1.60–1.90	1.61	1.45–1.74

**Table 3:** Spatiotemporal Parameters (non-normalized and normalized) for the two age subgroups

	Group A		Group B		
	6–8 years (*n* = 11)		9–10 years (*n* = 17)		*P*-value
	median	range	median	range	
	Non-Normalized				
Cadence (steps per second)	2.34	1.98–2.72	2.18	1.85–2.41	0.020*
Speed (ms^-1^)	1.20	0.88–1.45	1.35	1.15–1.55	0.002*
Step Length (m)	0.53	0.42–0.60	0.62	0.55–0.69	<0.001*
Stride Length (m)	1.03	0.82–1.17	1.23	1.09–1.36	<0.001*
	Normalized				
Cadence	0.91	0.77–1.11	0.79	0.68–0.93	0.535
Speed	0.45	0.36–0.59	0.49	0.44–0.58	0.129
Step Length	0.79	0.71–0.90	0.83	0.73–0.96	0.056
Stride Length	1.54	1.39–1.79	1.64	1.15–1.90	0.055

*significant difference

### Kinematic patterns and joint kinematics

**Fig. 1:**
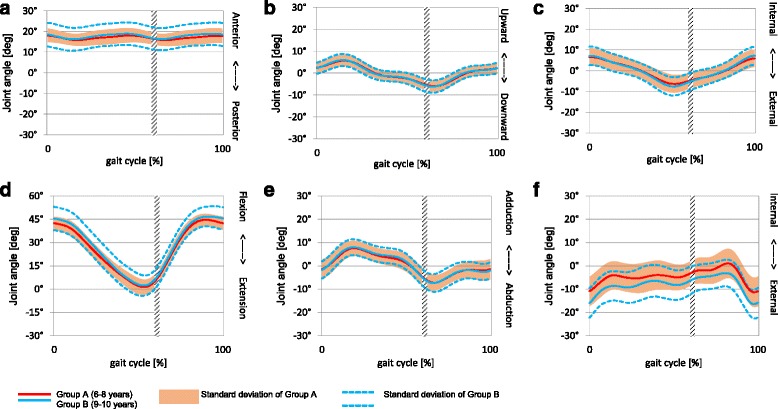
Kinematics of the two sub-groups: **a** Pelvis Tilt, **b** Pelvis Obliquity, **c** Pelvis Rotation, **d** Hip Flexion/Extension, **e** Hip Ab/Adduction, **f** Hip Rotation

**Fig. 2:**
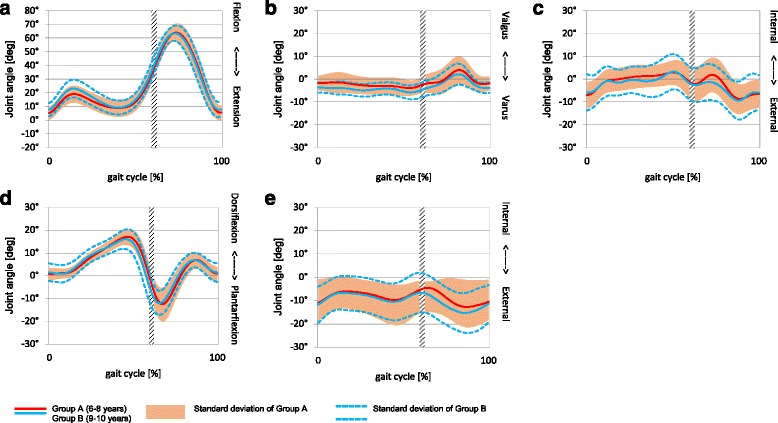
Kinematics of the two sub-groups: **a** Knee Flex/Extension, **b** Knee Ab/Adduction, **c** Knee Rotation, **d** Ankle Dorsi/Plantarflexion,** e** Foot Progression

The kinematic patterns of the pelvis, hip, knee, ankle and foot movements during a gait cycle are presented in Figs. 1 and 2. Pelvis tilt, hip F/E, knee F/E and ankle DF/PF occur in the sagittal plane; pelvis obliquity, hip abd/add and knee abd/add occur in the frontal plane; and pelvis rotation, hip rotation, knee rotation and foot progression occur in the transverse plane.

Figure 1 (a) – (e) shows minimum variation between the two age groups with a small standard deviation. However fig. 1 (f) demonstrates a larger variation between the two age groups.

Figure 2 (a), (b), and (d) also showed minimum variation, whereas fig. 2 (c) and (e) revealed a larger variation between the groups.

Tables 4 and 5 show the median and range values for the maximum and minimum values of the lower limb kinematics during the gait cycle respectively. There were no statistical differences between genders or between left and right sides for each of the joint angles. Thus, boys and girls and left and right sides were combined for the two age subgroups (Group A – 6–8 years; Group B – 9–10 years).

**Table 4:** The maximum values for the gait kinematics of the pelvis and lower limb kinematics for the two age groups

	Group A		Group B		
	6–8 years (*n* = 11)		9–10 years (*n* = 17)		*P* value
	median	range	median	range	
Pelvis X	18.2	12.5–24.9	19.5	7.0–28.6	0.495
Pelvis Y	6.0	3.4–8.6	5.7	2.6–9.4	0.944
Pelvis Z	6.6	4.0–11.9	8.1	3.4–11.7	0.249
Hip X	43.6	41.8–51.5	46.2	35.2–57.6	0.196
Hip Y	7.2	4.5–11.0	8.4	4.8–11.1	0.438
Hip Z	2.5	−5.1–10.0	0.01	−9.7–5.8	0.115
Knee X	65.0	54.7–71.3	64.8	50.6–70.5	0.906
Knee Y	4.9	−3.1–11.8	2.6	−2.5–10.6	0.312
Knee Z	6.8	−0.1–13.2	5.4	−8.9–21.5	0.384
Ankle X	16.5	12.4–20.5	16.4	6.6–23.1	0.557
Foot Progression Z	−1.8	−17.8–4.8	−4.1	−18.7–5.5	0.525

(X) Sagittal Plane; (Y) Frontal Plane; (Z) Transverse Plane

**Table 5:** The minimum values for the gait kinematics of the pelvis and lower limb kinematics for the two age groups

	Group A		Group B		
	6–8 years (*n* = 11)		9–10 years (*n* = 17)		*P* value
	median	range	median	range	
Pelvis X	15.2	11.0–20.4	16.4	3.0–24.3	0.495
Pelvis Y	−6.0	−8.7–−3.1	−5.9	−9.7–1.9	0.981
Pelvis Z	−6.6	−11.0–−3.3	−7.6	−11.2–2.5	0.115
Hip X	1.7	−5.8–8.4	2.9	−13.7–12.7	0.525
Hip Y	−7.7	−11.0–−3.4	−8.1	−13.1–2.8	0.981
Hip Z	−11.2	−19.9–−5.4	−17.0	−25.3–5.2	0.036*
Knee X	2.6	−2.6–9.4	6.5	−3.9–11.6	0.230
Knee Y	−4.7	−9.1–1.1	−7.1	−10.6–2.4	0.070
Knee Z	−11.3	−15.4–−3.8	−9.9	−23.6–3.6	0.869
Ankle X	−12.6	−22.4–−6.07	−11.8	−21.1–3.6	0.724
Foot Progression Z	−14.3	−25.9–−10.4	−16.0	−29.2–7.3	0.724

(X) Sagittal Plane; (Y) Frontal Plane; (Z) Transverse Plane

*significant difference

There was a statistical significant difference between the two groups for the mean hip rotation minimum values (*p* = 0.036), therefore Group B presented with more relative external rotation at the hip joint than Group A. There was no statistical significant difference between the two groups for any other kinematic parameter.

## Discussion

This is the first report on normative gait patterns of typically developed South African children. The findings of this study suggest that the kinematic patterns and spatiotemporal parameters of gait in typically developed children 6–10 years old are consistent with the published international literature which reported on the gait patterns of children in developed countries such as Australia, Norway, Germany, and China [11, 14, 34–36]. The results are also in agreement with recent studies indicating that there are no significant differences in spatiotemporal parameters or kinematics between genders [8, 14, 37]. Moreno-Hernández et al. [37] suggests that it is not until the adolescent years when neurological and musculoskeletal maturity is reached, that gender differences may be notable. Children reach adult-like sensory integration at the age of 12 years and may be gender specific [38]. Other studies have concluded that a child’s gait will continue to evolve in terms of spatiotemporal parameters (step and stride length, speed and cadence, balance and percentage of support) until a child is fully grown due to the changes in anthropometric measurements [39–41].

Chagas et al. [8] and Moreno-Hernández et al. [37] studied children between the ages of 6–13 years and reported a non-normalized mean cadence of 122.48 ± 13.83 steps/min and 117.9 ± 11.4 steps/min respectively. This compares well with our study. Our study has shown that non-normalized cadence was significantly lower (*p* = 0.02), the speed faster and the step and stride length longer for the older children (9–10 years) compared to the younger children (6–8 years). This concurs with Dusing and Thorpe [7] and Holm et al. [14] who also reported reduced cadence in older children compared to younger children. Consistent with the findings of the present study, Chagas et al. [8] and Moreno-Hernández et al. [37] reported no significant differences in normalized cadence when comparing age sub-groups of children. Comparisons between children and adults also revealed an on-going decrease in cadence as age increased [35, 42]. Cadence decreased with age when children, aged 5–13 years, were compared with young adults (mean age 19.7 years) [35]. Bovi et al. [42] compared children (6–17 years) with 20 adults and found no significant difference in cadence between the two groups. This could be since the younger group included adolescents, who already showed matured gait patterns and adult-like sensory-motor integration [13, 38]. Step and stride length increased with age, but was not significantly different between the two age sub-groups of our study. Both findings agree with published studies [7, 14, 34, 35, 42]. Non-normalized step and stride length increased with age, but normalized values remained unchanged [7, 14]. Although speed affects cadence, step length, stride length and other spatiotemporal parameters, as well as kinematics during gait, our study did not show a significant difference in walking speed between younger and older children [11, 15, 16, 43]. The median speed for the group in the current study compares well with international research based on Mexican children (6–13 years), Australian children (5–13 years) and American children (9–11 years) who walked at a self-selected speed of 1.13(±0.19) ms^-1^, 1.37(±0.17) ms^-1^ and 1.22 (±0.04) ms^-1^ respectively [35, 37, 44]. Thus, 6–10-year-old South African children’s spatiotemporal parameters of gait fall within the international norms when compared with those of other countries.

The kinematic gait patterns were similar between the younger and older children which could be attributed to negligible differences in walking speed, exposure to similar levels and type of physical activities and the absence of gross developmental or structural abnormalities in our participants [43]. We also noted similar peak hip, knee and ankle angles between the older and younger age groups. These differences in peak angles were from 0.1° up to 5.8° between the two age groups (see Table 4 and 5). Our finding compares to published reports [11, 13, 36].

Cigali et al. [12] and Shih et al. [36] reported similar mean peak hip abd/add angles in 50 children aged 7–11 years (−3.30 ± 2.32 – 6.33 ± 5.54) and 10 children aged 9.7 ± 0.9 years (0.42 ± 3.52 – 8.93 ± 4.39) respectively compared to our reporting of median values. Cigali et al. [12] also reported mean peak values for knee E/F (−7.06 ± 6.76 – 55.56 ± 3.11), ankle PF/DF (−21.85 ± 6.03 – 12.08 ± 12.27) and foot progression (−18.50 ± 11.80 – 11.00 ± 11.80) which falls within the standard deviation band width of our study (see Fig. 2). Shih et al. [36] reported comparable mean peak knee add/abd and knee external/internal rotation values of−2.21 ± 4.42 – 3.42 ± 4.89 and−10.18 ± 6.54 – 3.08 ± 5.07 respectively. The studies by Nikolajsen et al [44] and Kung et al. [45] reported only on the joint kinematics during the stance phase of children aged 10 years old and reported similar hip, knee and ankle mean peak values as see in our study for the stance phase. For example, Kung et al [45] reported mean peak knee F, ankle DF and PF of 41.74 ± 3.72; 10.18 ± 3.15 and−11.78 ± 5.14 respectively. This could indicate that the kinematic gait patterns of the pelvis and lower limb of 6–10-year-old children are established, comparable to the joint kinematics of children from other countries and mimic more the adult-like patterns observed by Sutherland et al. [13]. They evaluated the gait of 309 children ranging from the onset of walking to seven years of age. They found that between the age of 3.5–4 years, children achieve maturation of gait. In a later study, they concluded that growth alone can explain most changes throughout the rest of the growing years [41]. As children mature and grow, their leg length and body height increase, which directly affect the time-distance parameters of gait [41].

When comparing joint kinematics within the two age subgroups, hip rotation was significantly different between the groups (*p* = 0.036). Older children (Group B) presented with more external rotation at the hip joint than the younger children (Group A). Femoral anteversion and hip internal rotation are highly correlated and both reduce significantly with advancing age. Thus, our study supports the fact that as a child develops, the degree of anteversion of the femoral head decreases and causes the older child to walk with more relative external rotation of the hip than a younger child [46, 47]. The degree of hip internal rotation may indicate surgical intervention in children with pathological gait. Hip rotation kinematic patterns might be age specific and should be considered accordingly when interpreting gait analysis data.

The study was limited by small numbers in certain sub-groups such as the number of 6-year-old boys. Kinetics were also not included in this study, but we recommend that future studies include kinetics as it could add valuable information to the understanding and interpretation of the gait patterns in typically developed 6–10-year-old children in South Africa.

## Conclusion

This study evaluated the 3D kinematics and spatiotemporal parameters of gait in 28 typically developed 6–10-year-old South African children. It provides normative values for gait parameters that show that this South African gait analysis laboratory compares well with international gait laboratories and values can be used for comparison during gait analysis. The study’s findings concluded that normalized spatiotemporal parameters were similar between the two age groups and are consistent with the values of children from other countries. The joint kinematic values showed significant differences for hip rotation, indicating that older children had more external rotation than younger children.

## Abbreviations

3D: Three-dimensional
Abd: Abduction
Add: Adduction
DF: Dorsiflexion
E: Extension
Ext rot: External rotation
F: Flexion
Int rot: Internal rotation
PF: Plantar flexion
PIG: Plug-in-Gait model
SD: Standard Deviation
